# Late Onset of Cerebellar Abiotrophy in a Boxer Dog

**DOI:** 10.4061/2010/406275

**Published:** 2010-12-05

**Authors:** Sanjeev Gumber, Doo-Youn Cho, Timothy W. Morgan

**Affiliations:** Department of Pathobiological Sciences, School of Veterinary Medicine, Louisiana State University, Skip Bertman Drive, Baton Rouge, LA 70803, USA

## Abstract

Cerebellar abiotrophy is a degenerative disorder of the central nervous system and has been reported in humans and animals. This case report documents clinical, histopathological, and immunohistochemical findings of cerebellar abiotrophy in an adult Boxer dog. A 3.5-year-old, female, tan Boxer dog presented with a six-week history of left-sided head tilt. Neurological examination and additional diagnostics during her three subsequent visits over 4.5 months revealed worsening of neurological signs including marked head pressing, severe proprioceptive deficits in all the four limbs, loss of menace response and palpebral reflex in the left eye, and a gradual seizure lasting one hour at her last visit. Based on the immunohistochemical staining for glial fibrillary acidic protein and histopathological examination of cerebellum, cerebellar cortical abiotrophy was diagnosed. This is the first reported case of cerebellar abiotrophy in a Boxer dog to our knowledge.

## 1. Introduction

Cerebellar abiotrophy is a term used to describe premature degeneration of fully formed cerebellar neurons caused by an intrinsic metabolic defect [1]. Cerebellar abiotrophy has been described in dogs, cats, sheep, cattle, pigs, horses [2], and alpaca [3]. Canine cerebellar abiotrophy was first reported in the Kerry blue terrier and has since been characterized as an autosomal recessive inherited disorder in that breed [1]. Cerebellar abiotrophy differs from cerebellar hypoplasia, which involves abnormal development of germinal populations of neuroepithelial cells [1, 2]. Clinically, animals with cerebellar abiotrophy are normal at birth and develop progressive neurological deficits during the postnatal period. Histologically, cerebellar abiotrophies typically involve a primary degeneration or loss of Purkinje neurons, variable loss of granule cells, and cortical astrogliosis [4]. The following case describes clinical, histopathological and immunohistochemical features of a putative case of cerebellar abiotrophy in a 3.5-year-old Boxer dog.

## 2. Case History

A 3.5-year-old, intact female tan Boxer was referred to veterinary teaching hospital and clinic for a six-weeks history of left-sided head tilt. On physical examination, the dog had significant left head tilt, circling to the right, ataxia, and mild ventral strabismus in the left eye. Intracranial disease was suspected, but the possibility of middle ear infection was not completely excluded. A complete blood cell count (CBC), serum biochemical panel, computed tomography (CT), and cerebrospinal fluid (CSF) analysis were performed. Results of the CBC and serum biochemical panel were within reference intervals. CT scan of the brain revealed mild bilateral hydrocephalus, which was considered to be an incidental finding or associated with a space occupying lesion in the brainstem. Therefore, a complete examination of the brainstem with magnetic resonance imaging (MRI) was indicated. The owner declined MRI at that time. The dog had chronic otitis externa, and the middle ear canals were within normal limits. CSF analysis revealed 27 nucleated cells/*μ*L (reference interval: <5 cells/*μ*L) and total protein of 21 mg/dL (reference interval: <30 mg/dL). CSF analysis was interpreted as mild, mononuclear pleocytosis, which could indicate infectious, inflammatory, or neoplastic disease. Polymerase chain reaction (PCR) analysis performed on CSF was negative for encephalitis panel (Canine Distemper virus, West Nile virus, *Borrelia burgdorferi*, *Neospora hughesi* and *caninum*, *Toxoplasma gondii*, *Anaplasma phagocytophilum*, *Ehrlichia canis*, *Rickettsia* spp., and Pan Fungal). A presumptive diagnosis of inflammatory brain disease of unknown cause or possible neoplasia was made. The dog was discharged on an anti-inflammatory dose of prednisone and oral lomustine (CCNU), a chemotherapeutic agent. 

During the second visit, four months after initial presentation, the dog presented with worsened neurological signs. Neurological examination revealed head pressing, severe proprioceptive deficits in all the four limbs, marked reduction of menace response, and palpebral reflex in the left eye. A poor clinical prognosis was given for the dog due to the progressive nature of the neurological disease. The dog presented again after three weeks with a history of severe seizure activity of one-hour duration. The dog was unresponsive and unable to ambulate, and humane euthanasia was performed. Postmortem examination revealed mild degenerative changes on the right lateral aspect of T3 vertebra (Diskospondylitis). The cerebellum was grossly normal in size and shape. There was no gross evidence of hydrocephalus which was noted on CT. No other gross abnormalities were present. Representative tissue samples including brain and spinal cord were fixed in 10% neutral buffered formalin. The samples were routinely processed, paraffin-embedded, sectioned at 5 *μ*m, and stained with hematoxylin and eosin. 

**Figure 1:**
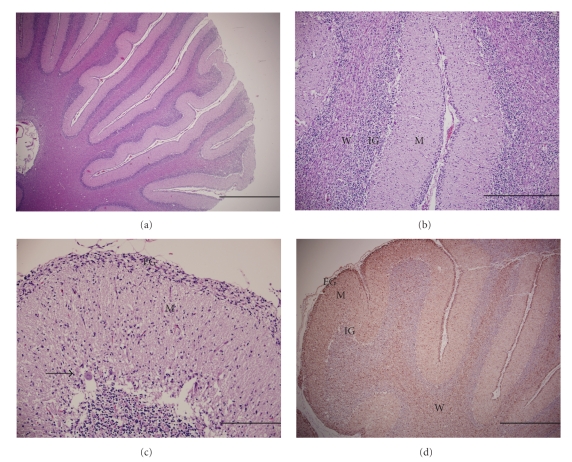
Cerebellum, dog. (a) Multifocal thinning of the cerebellar folia. Hematoxylin and eosin stain (HE). Bar: 1 mm. (b) Moderate hypocellularity of the inner granular layer (IG), increased cellularity in the white matter (W), and a well-defined molecular layer (M). HE stain. Bar: 500 *μ*m. (c) Moderate, multifocal depletion and degeneration of purkinje cells (arrow) and partial retention of the external granular layer (EG). HE stain. Bar: 200 *μ*m. (d) Marked astrogliosis evidenced by positive GFAP immunostaining in the external granular layer (EG), molecular layer (M), inner granular layer (IG), and white matter (W). Immunoperoxidase with 3-, 3′-diaminobenzidine with Mayer's hematoxylin counterstain. Bar: 1 mm.

Histologically, the cerebellar folia were irregularly slightly thinned, with moderate, multifocal depletion of Purkinje cells. The remaining Purkinje cells were widely separated, shrunken, hypereosinophilic with karyolysis (necrosis), and occasionally vacuolated (degeneration) (Figures 1(a) and 1(c)). The inner granular cell layer was moderately hypocellular (Figure 1(b)). There was a partial failure of migration of the external granular layer (Figure 1(c)). A marked increase in cellularity (astrogliosis) was present in the white matter, which was confirmed immunohistochemically. The sections of spinal cord had mild, multifocal axonal degeneration. The histopathological findings were consistent with cerebellar abiotrophy. 

Immunohistochemical staining of formalin-fixed, paraffin-embedded sections was performed as described by the manufacturer using glial fibrillary acidic protein (GFAP) rabbit polyclonal antibody (Code number Z0334) and myelin basic protein (MBP) rabbit polyclonal antibody (Code number 18-0038, EnVision+ System-HRP Labeled Polymer (DAB), Dako North America Inc., Carpinteria, CA.) The positive control tissue was a section of a cerebellum from an unaffected dog. The negative control tissue consisted of the same system without the primary monoclonal antibody. Immunohistochemical staining for GFAP revealed marked astrogliosis in the molecular and granular layers, white matter, and partially retained external granular layer (Figure 1(d)). MBP immunostaining showed no significant changes in the arborization of the fibers in the white matter. Marked astrogliosis on GFAP immunostaining further supported the histopathological diagnosis of cerebellar abiotrophy.

## 3. Discussion

Cerebellar abiotrophy has been identified in many breeds of dogs including rough-coated collie [5], Border collies [6], Australian kelpie [7], Labrador retrievers [8], Bernese mountain [9], Rhodesian ridgebacks [10], Portuguese podencos [11], Miniature Schnauzer [12], Scottish terrier [13], Beagle [14], English bulldog [15], and Lagotto Romagnolo [16]. The age of onset and progression of clinical signs of cerebellar dysfunction vary markedly according to the breed affected. The clinical signs associated with abiotrophic diseases are usually seen within first weeks or months of life and involve the cerebellar cortex [2]. Gordon setters [17] and Brittany spaniels [18] have been identified with onset of clinical signs from 6 to 30 months and 7 to 13 years of age, respectively. A similar late onset of disease was observed in the present case. Additionally, this disease has not been reported in a Boxer dog previously. 

The cause of cerebellar abiotrophy is currently unknown, but it is presumed to be an inherited genetic abnormality. It is thought to involve the excitotoxic degeneration of neurons containing glutamate receptors [1, 4]. A possible role of channelopathies with abnormal structure and function of voltage-gated calcium channels [19] and autoantibody-mediated disorders in humans has been suggested [20]. Canine herpesvirus has a predilection for the germinal layers of the cerebellum in puppies. Puppies that survive canine herpes virus infection have reduced number of cells in the granular layer and mild loss of Purkinje cells. These changes are consistently accompanied by foci of microgliosis, mononuclear cell infiltration, malacia and, mineralization [2, 21]. Such changes were not identified microscopically or with ancillary diagnostic tests in the current case. 

Histologically, there was a partial impairment of cerebellar granule cell migration along with cerebellar abiotrophy in the present case. In the developing central nervous system, immature cerebellar granule cells migrate from the external germinal layer through the molecular layer to form the inner granule cell layer [22]. Cortical granule cells that do not migrate to the inner granule layer typically fail to mature properly. Deficits in neuronal migration have been reported in humans following genetic mutation and exposure to environmental toxins. However, the exact pathophysiology of these cortical abnormalities is still unclear [23]. The likely cause of impaired neuronal migration in this case could be a genetic mutation. 

MRI findings are normal during early stages of cerebellar abiotrophies, but late stages of chronic abiotrophy reveal atrophy of the cerebellar folia [4]. MRI might have detected the cerebellar abiotrophy in the present case; however, the owner declined further diagnostics at the initial visit. MRI has been used successfully for the diagnosis of cerebellar cortical degeneration in a Scottish terrier [13]. Reduction of cerebellar size has been identified in several reports of cerebellar abiotrophies [11, 24, 25]. No gross changes were detected in the cerebellum during postmortem examination in this dog although the clinical history was compatible with chronic progression of cerebellar abiotrophy. The absence of gross changes could be due to breed predisposition; however, a specific cause remains undetermined. To the authors' knowledge, this is the first reported case of cerebellar abiotrophy in a Boxer dog. Additional cases and further research are required to prove heritability in this breed although the authors were unable to completely determine the pure-bred status of the animal.
